# [Corrigendum] miR‑195 inhibits cell proliferation and angiogenesis in human prostate cancer by downregulating PRR11 expression

**DOI:** 10.3892/or.2023.8507

**Published:** 2023-02-22

**Authors:** Chao Cai, Huichan He, Xiaolu Duan, Wenqi Wu, Zanlin Mai, Tao Zhang, Junhong Fan, Tuo Deng, Wen Zhong, Yongda Liu, Weide Zhong, Guohua Zeng

Oncol Rep 39: 1658–1670, 2018; DOI: 10.3892/or.2018.6240

Subsequently to the publication of the above paper, an interested reader drew to the authors' attention that a pair of data panels featured in Figs. 1B and 4C contained overlapping sections, such that data that were intended to show the results from differently performed experiments appeared to have been derived from the same original source (specifically, the ‘LNCaP / miR-NC’ panel in Fig. 1B and the ‘LNCaP / miR-195+PRR11’ panel in Fig. 4C were overlapping).

The authors were able to re-examine their original data files, and realized that this figure had been inadverently assembled incorrectly. The revised version of Fig. 1, containing the correct data for Fig. 1B (wherein the error was contained), is shown on the next page. Note that the revisions made to this figure do not affect the overall conclusions reported in the paper. The authors are grateful to the Editor of Oncology Reports for allowing them the opportunity to publish this Corrigendum, and apologize to the readership for any inconvenience caused.

## Figures and Tables

**Figure 1.**
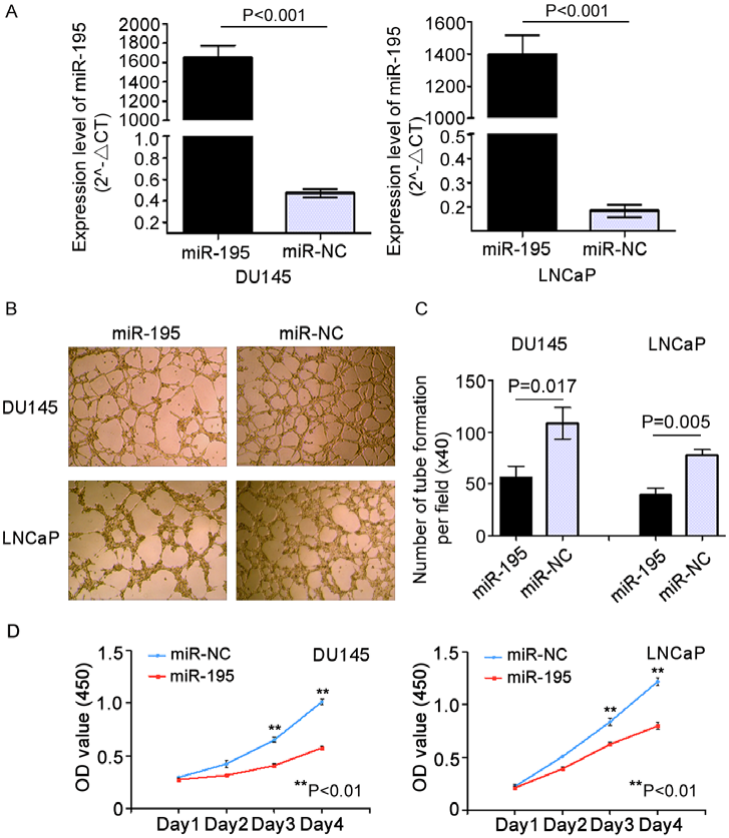
Overexpression of miR-195 suppresses the proliferation of LNCaP and DU145 cells and inhibits human umbilical vein endothelial cell (HUVEC) tube formation *in vitro*. (A) miR-195 levels were determined by RT-qPCR at 48 h after transient transfection of DU145 and LNCaP cells with miR-195 mimics. miR-195 expression (miR-195/U6) was calculated as fold change relative to the negative control (NC). (B) HUVEC tube formation was inhibited by the addition of conditioned media from DU145 and LNCaP cells transfected with miR-195 mimics. (C) The number of complete tubes per field (×40) was calculated. (D) Enforced expression of miR-195 inhibited the proliferation of DU145 and LNCaP cells. Statistical analysis was performed on the results from three independent experiments. Data are presented as means ± SD. **P<0.01 compared with the negative control.

